# Cheoplastic Method of Mounting Artificial Teeth

**Published:** 1859-01

**Authors:** A. A. Blandy

**Affiliations:** Baltimore


### ARTICLE XI.

### *Cheoplastic Method of Mounting Artificial Teeth.*

Messrs. Editors:

Absence from the United States caused the neglect of communications, due from me to the profession, in relation to several articles whose object has been the abuse of Cheoplasty. As has been remarked elsewhere, the subject is not one for controversy. I can only state that facts admit of no denial, and although there may be those found, who for weak motives, may attempt to impugn the assertion and honesty of those who have stated such facts, still such remain as undoubted evidences of the validity of all claims made for Cheoplasty. I will agree to show teeth mounted by my process which has been in practical use, ranging from six months to two years, for every line that has been written derogatory of it, and I doubt not, by visiting a few gentlemen of large practice using it, I could show a piece for each word.

What can divers opinions do in the transformation of these results, and what possible use can be the encouragement of abuse by parties who, of themselves, have had no experience or acquaintance with it, whatever, and perhaps little in any other.

Some pretend to know what metals enter into my alloy and give my careful admonitions, which would be, *perhaps*, good, if the metals of their compound entered into mine. I beg, in conclusion, to announce through your Journal, that Cheoplasty has only become more valuable and more acceptable, by its use, in the hands of several hundred respectable practitioners, from its economy, practical usefulness, lightness, strength, purity, and vast saving in time.

Baltimore, Dec. 4, 1858. A. A. BLANDY.

